# *Vincetoxicum samothracicum* (Apocynaceae), a new species from Mt. Saos, Samothraki Island, Greece

**DOI:** 10.3897/phytokeys.270.174922

**Published:** 2026-01-28

**Authors:** Katerina Goula, Panagiotis Doumas, Theophanis Constantinidis

**Affiliations:** 1 Section of Ecology & Systematics, Department of Biology, National and Kapodistrian University of Athens, Panepistimiopolis, 15784 Athens, Greece National and Kapodistrian University of Athens Athens Greece; 2 8th Elementary School, Xanthi, Greece 8th Elementary School Xanthi Greece

**Keywords:** Asclepiadoideae, Greek endemic, ITS, Mediterranean area, North Aegean, phylogenetic analysis, taxonomy

## Abstract

Samothraki, an island of the North Aegean region of Greece, hosts a remarkable flora that includes seventeen endemics restricted to its boundaries and several species of phytogeographical importance. A floristic investigation of the upper parts of the island, on Mt. Saos, revealed populations of a reddish-purple-flowered *Vincetoxicum* morphologically distinct from any species known from Greece and neighboring countries. This new species, named *Vincetoxicum samothracicum*, is apparently related to a group of east-Mediterranean species with corona lobes free almost to the base, but it differs from all of them in the characters of the flowers, fruits, and indumentum. Investigation of an internal transcribed spacer (ITS) *Vincetoxicum* phylogeny did not place the new species in the same monophyletic group as its presumed morphological relatives. *V. samothracicum* is a local species, but it is currently assigned to the IUCN category LC.

## Introduction

The genus *Vincetoxicum* Wolf, in its broad circumscription, is among the largest and most widespread genera of Apocynaceae, subfamily Asclepiadoideae (Güven et al. 2019). It comprises more than 200 species (Kidyoo et al. 2023), distributed in Africa, Asia, and Europe, with several species introduced into North America (Liede-Schumann et al. 2016). Taxonomically, *Vincetoxicum* is complex, as reflected in its intricate nomenclatural history (Liede-Schumann and Meve 2018). All *Vincetoxicum* species occurring in Europe that have been sampled in molecular phylogenetic studies belong to the *Vincetoxicum* s. str. clade, a temperate clade extending from Japan to western Europe (Liede-Schumann et al. 2016; Güven et al. 2019). A high degree of uniformity has been observed in the vegetative morphology of closely related *Vincetoxicum* species (Shah et al. 2021), and within *Vincetoxicum* s. str., several taxa further exhibit rather similar floral characteristics (Liede-Schumann et al. 2016). This morphological similarity often complicates species identification and the delimitation of infrageneric entities. Previous studies indicated that morphology alone has limitations in addressing species concepts and relationships within *Vincetoxicum*; subsequently, molecular phylogenetic approaches have made a significant contribution to defining boundaries among groups and species within the genus (Liede-Schumann et al. 2016; Shah et al. 2021). A need for regional taxonomic revisions within the genus has been highlighted (Liede-Schumann et al. 2012, 2016; Shah et al. 2021).

In Greece, seven species and nine taxa of *Vincetoxicum* have been reported so far, distributed across all floristic regions except the Kiklades Islands (Kik, Dimopoulos et al. 2024). The only species endemic to the country is *V. creticum* Browicz, restricted to the mountains of eastern and western Kriti (Strid 2024a). Four additional taxa have a limited distribution in Greece: *V. huteri* Vis. & Asch. is confined to Kerkira Island (Hansen 1980); *V. funebre* Boiss. & Kotschy has been reported from Mt. Timfristos, although its presence there, and in Greece as a whole, is highly doubtful (Strid 2024b); *V. canescens* subsp. *pedunculatum* Browicz occurs on the East Aegean Islands of Lesvos, Chios, Samos, and Ikaria; and *V. canescens* (Willd.) Decne. subsp. canescens has been recorded only on Rodos Island (Strid 2024a).

The island of Samothraki is located in northeastern Greece, surrounded by the North Aegean Sea. Together with Thasos, Limnos, and Agios Efstratios, it forms the floristic region of the North Aegean Islands (NAe; Strid and Tan 1997). An assemblage of 1441 species has been reported from Samothraki so far, including seventeen endemics restricted to the island (Biel and Tan 2014). For *Vincetoxicum*, Biel and Tan (2014) identified only *V. fuscatum* (Hornem.) Rchb. on the island, whereas Strid (2016b, 2024a) additionally reported *V. hirundinaria* subsp. *nivale* (Boiss. & Heldr.) Markgr. and *V. speciosum* Boiss. & Spruner.

In 2015, and again in 2018, the mountaineer Eva Arampatzi and Dr. Konstantinos Touloumis sent photographs of an unusual *Vincetoxicum* species from Mt. Saos, Samothraki, to the first author. Independently, the second author had located the same plant in the summit area of Mt. Saos as early as 2012. The distinctive reddish-purple flowers and fruits, unique among the Greek species, intrigued the first two authors. In 2021, they ascended the mountain together to collect samples and investigate the species’ identity in detail. All authors soon realized that a new species was involved, and this study presents the results of its taxonomic and molecular investigation. The new species is herein described as *Vincetoxicum samothracicum*.

## Methods

Plant material was collected from the summit area of Mt. Saos, Samothraki, in the summer of 2021. The samples were used for the preparation of herbarium specimens, which are deposited in ATHU and ATH (acronyms follow Thiers 2022, continuously updated). The morphology of the plant was studied and compared with different but related *Vincetoxicum* taxa, using descriptions published in relevant literature sources (Pobedimova 1952; Rechinger 1970; Markgraf 1972; Browicz 1975, 1978; Strid 1991, 2016a). In addition, specimens and digital images of specimens provided by the herbaria ATH, ATHU, B, BR, E, GOET, K, MO, P, S, US, and WAG were examined.

For the molecular investigation, DNA was extracted from dried leaves using the NucleoSpin® Plant II kit (Macherey-Nagel). The universal primers ITS4 and ITS5 (White et al. 1990) were used for amplification of the nuclear ITS region. Polymerase chain reaction (PCR) was carried out in a 50 μL reaction volume containing 5 μL of polymerase reaction buffer, 4 μL of MgCl_2_, 3 μL of dNTPs, 2 μL of each primer, 0.4 μL of Taq DNA polymerase (KAPA HiFi Roche™), 2 μL of template DNA, and 31.6 μL of ddH_2_O. Thermal cycling conditions were as follows: initial denaturation at 94 °C for 3 min; 35 cycles of denaturation at 94 °C for 1 min, annealing at 55 °C for 1 min, and extension at 72 °C for 3 min; followed by a final extension at 72 °C for 10 min. The PCR product was sequenced with the same primers using the Sanger sequencing method. The new sequences were deposited in GenBank (https://www.ncbi.nlm.nih.gov/) under the accession numbers PX457925–PX457926 (ITS5–ITS4r; release date 31 March 2026, if not published earlier). Additional sequences used in the phylogenetic analysis included 52 *Vincetoxicum* accessions, representing 27 taxa, as well as accessions from two outgroup genera of the Asclepiadoideae, all retrieved from the same repository (Suppl. material 1). Chromatogram quality control and sequence alignment were performed using CodonCode Aligner v. 11.0.2. The Nexus file used in the phylogenetic analyses is provided in Suppl. material 2. Bayesian inference (BI) analysis was conducted in MrBayes v. 3.2.7a (Ronquist et al. 2012). The Markov chain Monte Carlo (MCMC; Altekar et al. 2004) algorithm performed four simultaneous runs for 5,000,000 generations, saving one tree every 5,000 generations, with the first 25% discarded as burn-in. Maximum likelihood (ML) analysis was carried out in IQ-TREE v. 1.6.12 (Nguyen et al. 2015). Phylogenetic analysis was performed using the TN+F+G4 model as implemented in IQ-TREE. Each branch in the phylogenetic tree was annotated with two support values: the first representing support calculated using SH-aLRT and the second obtained using the ultrafast bootstrap approximation (UFBoot; Hoang et al. 2018), implemented in IQ-TREE, with 1000 bootstrap replicates. Trees were visualized in FigTree v. 1.4.4 (Rambaut 2018).

The distribution map was created using the free and open-source software QGIS 2025 (v. 3.42.3). The extent of occurrence (EOO) and area of occupancy (AOO) were evaluated using GeoCat (2025), and the threat status was assessed in accordance with the IUCN Red List Categories and Criteria (IUCN Standards and Petitions Committee 2024).

## Results

### Morphology

**Figure 1.**
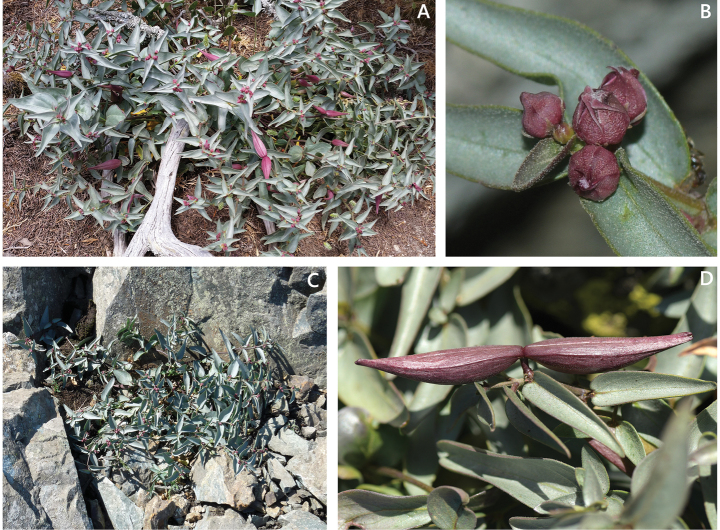
*Vincetoxicum samothracicum* on Mt. Saos, Samothraki. **A**. Plant growing on granitic substrate, next to a *Juniperus oxycedrus* chump; **B**. Inflorescence; **C**. Plant growing near the summit on diabase basalt; **D**. Mature follicles. Photos by K. Touloumis (**A**), K. Goula (**B, D**), and P. Doumas (**C**).

The *Vincetoxicum* plants from the upper parts of Samothraki stand out at first glance due to the unusual color of their flowers and fruits (Fig. 1). Species distributed in Greece may have brown or brownish-purple flowers, but those with strictly reddish-purple flowers are absent (Markgraf 1972; Browicz 1978; Strid 1991, 2016a, 2024a). Plants with flowers in shades of red, rather than brown, are very rare in Europe (Markgraf 1972) and Turkey (Browicz 1978; Güven et al. 2021). The only species with a reddish corolla previously reported in the *Vincetoxicum* s. str. clade is *V. rossicum* (Kleopow) Barbar. (Pobedimova 1952), which differs fundamentally from the Samothraki plants, i.e., in the morphology and pubescence of the stem, leaves, and corolla. *V. rossicum* has a single, twining, biserially pubescent stem, leaves 6–8 × 3–4 cm, paler on the abaxial side, and a corolla that is glabrous on the adaxial side (Pobedimova 1952), whereas the plants from Samothraki have numerous stems that are puberulent all around, leaves smaller than 6 × 2.6 cm and concolorous on both sides, and a corolla that is lanate on the adaxial side.

**Figure 2.**
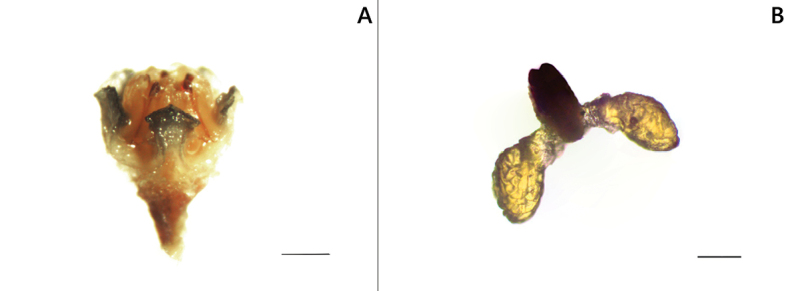
*Vincetoxicum samothracicum*. **A**. Gynostegium showing deltoid corona lobes free almost to their bases; **B**. Pollinarium. Scale bars: 1 mm (**A**); 100 μm (**B**).

The corona of the Samothraki plants has lobes free almost to their base (Figs 2A, 5F), a feature common to a small group of species distributed in the eastern Mediterranean region, namely *Vincetoxicum canescens*, *V. creticum*, and *V. tmoleum* Boiss. (Güven et al. 2019). The first two species are also present in Greece. According to Güven et al. (2019), coronas with lobes free almost to their base can also be found in certain species within *Vincetoxicum* s. str. that are usually distributed outside the Mediterranean region. Of these, only *V. parviflorum* Decne. occurs in Turkey, with the remaining species distributed further east. Despite the similarity in the corona type, there are significant morphological differences between all these species and the plants growing in the upper parts of Samothraki (Rechinger 1970; Browicz 1978; Ali and Khatoon 1982; Zaeifi 1999). Furthermore, all species reported from Samothraki so far (*V. fuscatum*, *V. hirundinaria* subsp. *nivale*, and *V. speciosum*; Biel and Tan 2014; Strid 2024a) have coronas with at least partly fused lobes (Güven et al. 2019).

Significant morphological similarity was observed among the eastern Mediterranean species *Vincetoxicum canescens*, *V. creticum*, *V. tmoleum*, and *V. samothracicum*. All these taxa differ from the remaining Greek *Vincetoxicum* species in the corona (lobes free almost to their base vs. connected), the pubescence on the adaxial side of the corolla (densely hairy vs. glabrous to pubescent), the shapes of the pollinium (ovoid vs. obovoid or clavate), and the corpusculum (ovoid vs. oblong) (Güven et al. 2015, 2019; our observations). To facilitate comparison, the taxonomically important differences between *V. canescens*, *V. creticum*, *V. tmoleum*, and *V. samothracicum* are summarized in Table 1.

**Table 1.** Differences between *Vincetoxicum samothracicum*, its most similar species (*V. canescens*, *V. creticum*, *V. tmoleum*), and the taxa (*V. fuscatum*, *V. hirundinaria* subsp. *nivale*, *V. speciosum*) reported from the island (Degen 1891; Ade and Rechinger 1938; Biel and Tan 2014; Strid 2016b, 2024a). Descriptions and measurements follow Markgraf (1972), Browicz (1978), Strid (1991, 2016a), and our observations.

Character	*Vincetoxicum samothracicum*	*V. canescens*	*V. creticum*	*V. tmoleum*	*V. fuscatum*	*V. hirundinaria* subsp. *nivale*	*V. speciosum*
Habit	procumbent	procumbent	procumbent	erect	erect	erect	erect
Shape of upper leaves	lanceolate to narrowly lanceolate, often obtuse	ovate, acute	lanceolate to ovate, acute	lanceolate to ovate, acute	lanceolate to ovate, acute	lanceolate to ovate, acute to acuminate	ovate to broadly lanceolate, acute
Petiole length	leaves sessile or petioles up to 1 mm	leaves subsessile	leaves subsessile	up to 10 mm	3–8(–10) mm	5–10 mm	leaves subsessile
Abaxial leaf pubescence	crisped pubescent to glabrescent	grayish tomentose	subglabrous	crisped pubescent	puberulent on veins and margins	puberulent on veins and margins to subglabrous	velutinous, especially on the veins
Stem pubescence	crisped puberulent all around	grayish tomentose all around	subglabrous	crisped pubescent all around	pubescent on two lines	pubescent on two lines to subglabrous	velutinous all around
Number of flowers per inflorescence	(1–)3–5(–6)	5–12	2–5	3–9	(3–)4–6(–8)	3–10	5–15
Sepals	lanceolate	ovate-lanceolate	ovate-lanceolate	lanceolate	narrowly ovate to lanceolate	narrowly lanceolate	narrowly lanceolate
Corolla color on the adaxial side	reddish purple	dull yellow	dull yellow	greenish yellow	yellowish-brown to dark purplish-brown	cream to pale greenish-yellow	dark to blackish-purple
Indumentum on the adaxial side of the corolla	lanate	hirsute	hirsute	lanate	glabrous or pubescent	glabrous	pubescent
Follicle shape	lanceolate	ovoid, acuminate	lanceolate, slender	lanceolate, slender	lanceolate, slender	lanceolate	lanceolate, slender
Follicle color	reddish purple	green	greenish yellow	greenish yellow	light brown	light brown	not seen
Follicle pubescence	minutely puberulent to glabrescent	gray puberulent	minutely puberulent to glabrescent	glabrous	glabrous	glabrous	velutinous

When *Vincetoxicum samothracicum* is compared with those *Vincetoxicum* species previously reported from Samothraki (Degen 1891; Ade and Rechinger 1938; Biel and Tan 2014; Strid 2016b, 2024a), several morphological differences emerge, apart from the corona type. The main distinguishing features between *V. samothracicum* and the species reported from Samothraki are summarized in Table 1.

### Phylogenetic analyses

**Figure 3.**
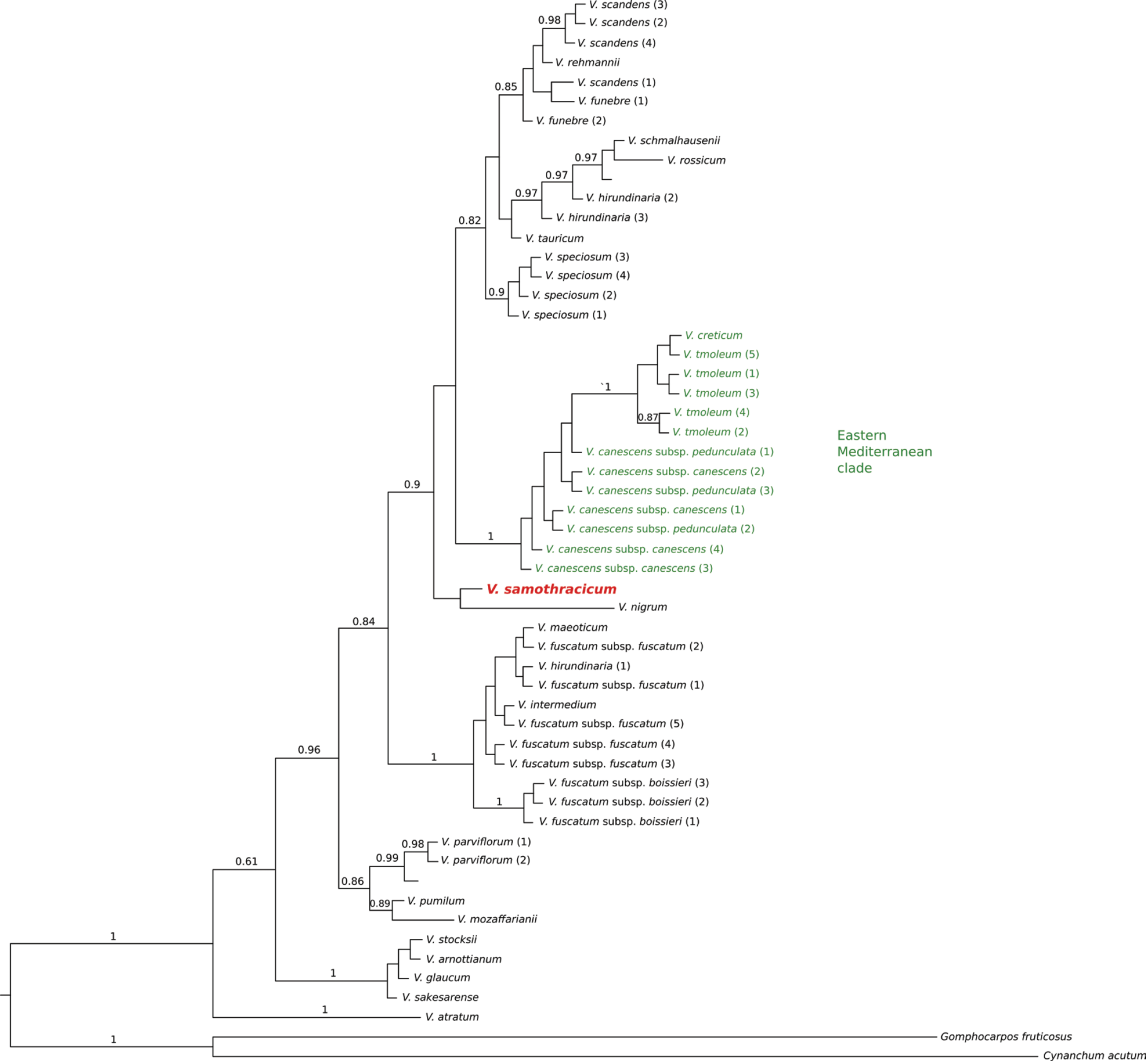
Bayesian inference phylogenetic tree of *Vincetoxicum*, based on the ITS region of 27 species of the genus and two Asclepiadoideae outgroups. Posterior probabilities above 0.6 are indicated on the branches.

**Figure 4.**
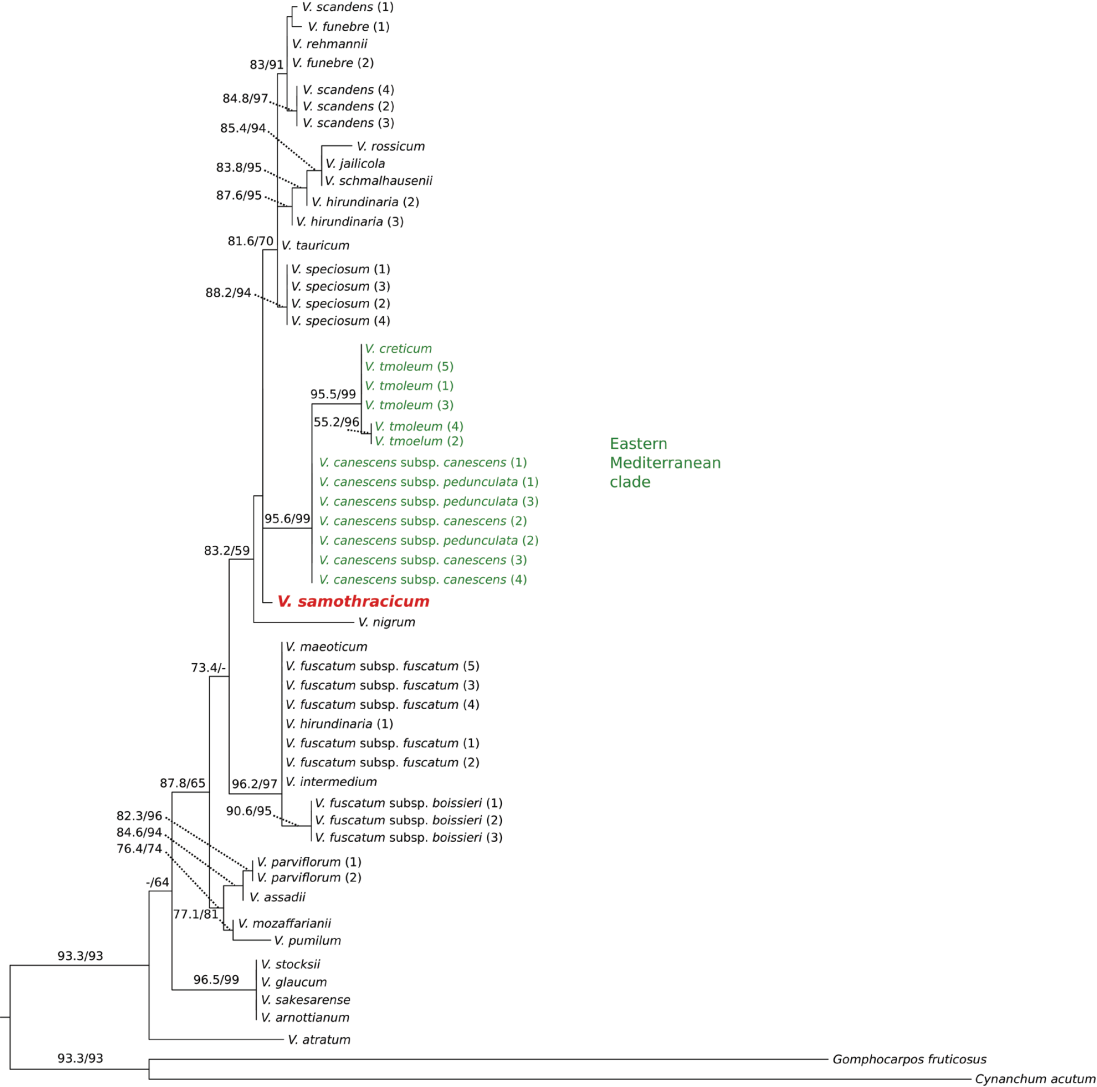
Maximum likelihood phylogenetic tree of *Vincetoxicum*, based on the ITS region of 27 species of the genus and two Asclepiadoideae outgroups. Numbers on the branches indicate SH-aLRT support (left) and UFBoot support (right). Only values above 50 are shown.

The phylogenetic trees generated using Bayesian inference (BI; Fig. 3) and maximum parsimony (MP, Fig. 4) exhibited different topologies; however, no strongly supported conflicting relationships were observed between them. As indicated in earlier studies, the phylogenetic trees demonstrate that all examined *Vincetoxicum* taxa form a monophyletic group, clearly separated from the outgroup (Liede-Schumann et al. 2016; Güven et al. 2019, 2021; Kidyoo and Kidyoo 2023). Both trees recovered a well-supported clade that includes *Vincetoxicum canescens*, *V. creticum*, and *V. tmoleum*, all belonging to the “Eastern Mediterranean clade” (see also Liede-Schumann et al. 2016; Güven et al. 2019). This clade represents the group of taxa most closely related morphologically to *V. samothracicum*. Nevertheless, the representative sample of *V. samothracicum* does not fall within the Eastern Mediterranean clade but is placed in an unresolved position adjacent to it.

**Figure 5.**
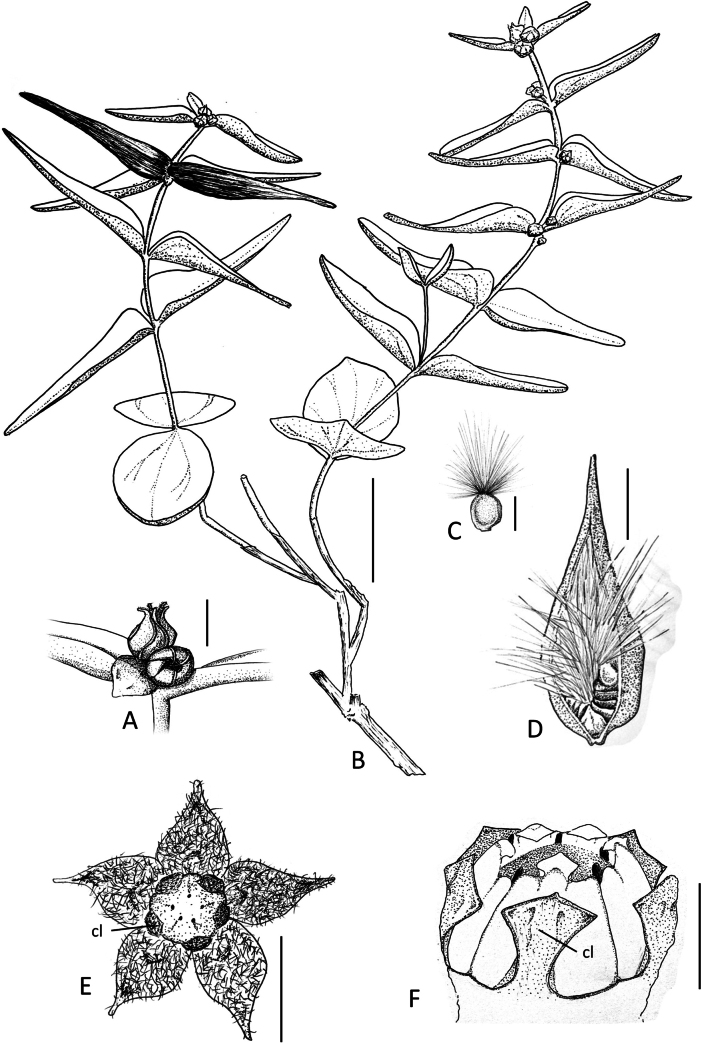
*Vincetoxicum samothracicum* Goula, Doumas & Constantinidis drawn by N.A. Katsaros from the holotype (Goula & Doumas 3225), ATHU. **A**. Flowers; **B**. Flowering and fruiting branches; **C**. Seed; **D**. Dehiscing follicle; **E**. Flower opened up to show indumentum inside and gynostegium; **F**. Gynostegium. Corona lobes (cl) are indicated in (**E**) and (**F**). Scale bars: 4 mm (**A, E**); 4 cm (**B**); 5 mm (**C**); 1 cm (**D**); 1 mm (**F**).

## Discussion

*Vincetoxicum samothracicum* is morphologically clearly placed within the species group that constitutes the “Eastern Mediterranean clade” of the genus, as presented by Liede-Schumann et al. (2016), i.e., *V. canescens*, *V. creticum*, and *V. tmoleum*. This group comprises closely related species, as indicated by morphological (Browicz 1975; Güven et al. 2015), karyological (Güven et al. 2019), palynological (Güven et al. 2019), and molecular (Liede-Schumann et al. 2016; Güven et al. 2019, 2021; this work) evidence. The morphology of *V. samothracicum*, particularly the corona with lobes free almost to their base, corolla pubescence, and the shape of the pollinium and corpusculum, clearly relates this species to the above-mentioned group. Yet, the unique corolla color, upper leaf shape, and additional morphological features presented in Table 1 differentiate it well from all members of the group. In our molecular ITS phylogenetic analyses, members of the Eastern Mediterranean clade form a clearly defined clade with high support (posterior probability: 1; bootstrap values: 95.6/99). The Samothraki specimen analyzed is not nested within this clade according to our data, and its phylogenetic position remains unresolved. Our results support the recognition of a new, distinct species described below as *Vincetoxicum samothracicum* Goula, Doumas & Constantinidis, sp. nov.

Previous floristic publications (Degen 1891; Ade and Rechinger 1938; Biel and Tan 2014; Strid 2016b, 2024a) did not agree on the number and identity of the *Vincetoxicum* species occurring on Samothraki. Biel and Tan (2014) reported *V. fuscatum* as the only species present on the island. The photograph in their book (p. 211), however, clearly depicts *V. samothracicum*. Nevertheless, *V. fuscatum*, as well as *V. hirundinaria* and *V. speciosum* (Strid 2016b, 2024a), may also occur on Samothraki.

The island of Samothraki hosts a remarkably high number of endemics restricted to its boundaries, especially when compared with other Greek islands of similar size. Samothraki has an area of 178 km^2^ and supports eighteen single-island endemics (SIEs), including *Vincetoxicum samothracicum*. In contrast, Skiros (209 km^2^) has only two SIEs; Milos (151 km^2^) and Tinos (194 km^2^) have only one each, whereas Paros (196.3 km^2^) hosts none (Strid 2016a; Goula and Constantinidis 2021). Thasos and Limnos, members of the same floristic region of the North Aegean Islands, have five and one SIEs, respectively (Strid 2016a). This high number of SIEs on Samothraki is consistent with its geographical position, degree of isolation, and distinctive geology and geomorphology (Kotopouli et al. 1989; Georghiou and Delipetrou 2010; Biel and Tan 2014; Kougioumoutzis et al. 2021).

### Taxonomic treatment

#### Vincetoxicum samothracicum

Taxon classificationPlantaeGentianalesApocynaceae

Goula, Doumas & Constantinidis sp. nov.

DDE3F748-213E-5EB4-8596-78C0F5B3D58C

urn:lsid:ipni.org:names:77375732-1

##### Diagnosis.

Related to but distinct from *Vincetoxicum canescens* by its unique reddish-purple color of the corolla and follicles (vs. dull yellow and green, respectively), the narrowly lanceolate, often obtuse upper leaves (vs. ovate, acute), and the lanceolate follicles (vs. ovoid). Also related but differing from *V. creticum* and *V. tmoleum* in a number of morphological characters (Table 1).

##### Type.

Greece • Samothraki Island: Nomos Evrou, Eparchia Samothrakis. Mt. Saos, along the main path from the Therma settlement to the mountain’s summit, ca. 880 m linear distance NW of the summit, rocky slopes, diabase basalt, 1383 m a.s.l., 40°28'N, 25°35'E, 28 July 2021, *Goula & Doumas 3225* (holotype, ATHU; isotype, ATH). Figs 1, 2, 5.

##### Description.

Perennial multibranched procumbent herb ca. 30–70 cm broad, stems 15–35(–40) cm long, stem and branches hollow, grayish-green to purple, puberulent with crisped, deflexed hairs. ***Leaves*** grayish-green; lower leaves (25–)35–60 × 18–26 mm, ovate to broadly ovate, broadly cuneate to rounded or subcordate, often oblique at base, subacute, sessile to subsessile, glabrescent, crisped-puberulent only at margins and veins on both sides; upper leaves gradually smaller, lanceolate to narrowly lanceolate, cuneate to rounded or subcordate at base, subacute or obtuse, subsessile or with a petiole up to 1 mm long, crisped-puberulent at margins and veins to glabrescent on both sides. ***Inflorescences*** extra-axillary, sessile, (1–)3–5(–6) flowered; pedicels 1–1.5 mm long and 0.2 mm wide. ***Sepals*** lanceolate, acute, ca. 1.5 mm long, glabrescent, green with narrow white, ciliate margins. ***Corolla*** reddish-purple, lobes contorted in bud and apparently remaining erect, forming an urceolate flower, ca. 4 × 2 mm, broadly lanceolate, acuminate, abaxially almost glabrous, adaxially with dense, white lanate hairs up to 0.5 mm long. ***Corona*** of five erect segments free almost to their base; each segment ca. 1 mm long, broadly deltoid apically becoming narrower towards the base, margins and apex slightly recurved. ***Pollinarium*** with pollinia ovoid, ca. 195–210 μm long; corpusculum ovoid to oblong, ca. 210–240 μm long. ***Follicles*** 4–5 × ca. 1 cm, lanceolate, minutely puberulent to glabrescent, reddish-purple. ***Seeds*** 5–6 × ca. 4 mm, brown to reddish-brown, slightly winged along margins; coma white, 12–14 mm long.

##### Distribution.

**Figure 6.**
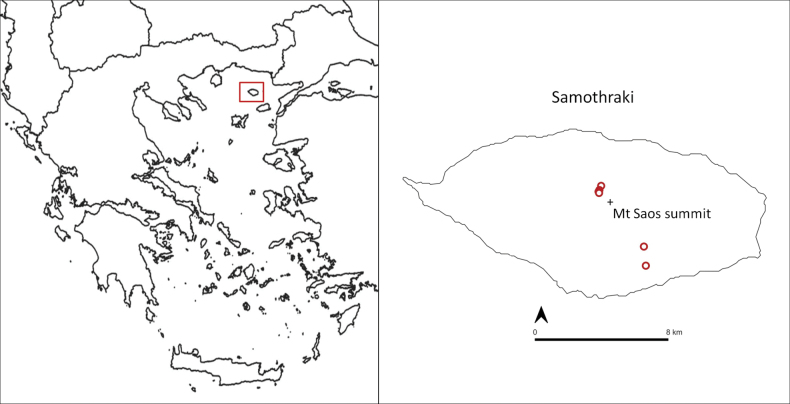
Distribution map of *Vincetoxicum samothracicum* on Samothraki, in the northeastern part of the Aegean Sea, Greece.

At present, *Vincetoxicum samothracicum* is known only from Mt. Saos, Samothraki, where it is restricted to two distinct areas (Fig. 6). The larger of these is located on the northern slopes of the mountain, close to the main summit, at 1383–1555 m a.s.l. Individuals have also been recorded on the southeastern slopes of the mountain, ca. 6 km SSE of the former area, at approximately 780–1045 m a.s.l. Biel and Tan (2014) reported *V. fuscatum* from the estuary of the Vatos stream, near sea level, but the identity of those plants remains unknown.

##### Ecology and habitat.

**Figure 7.**
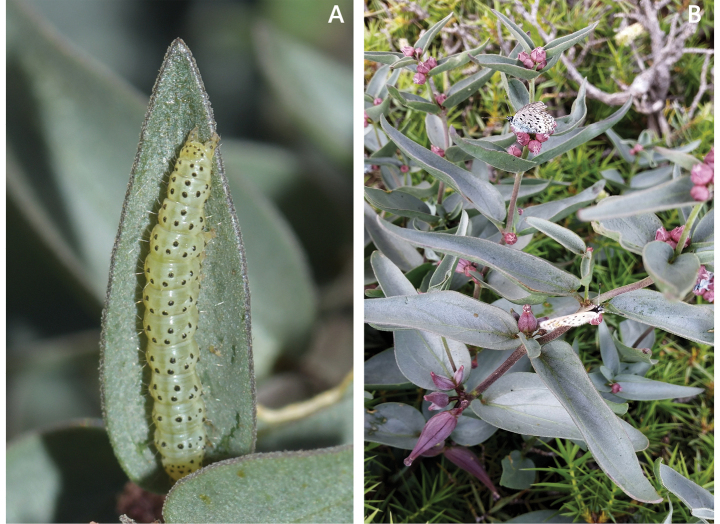
Lepidoptera observed on *Vincetoxicum samothracicum*. **A**. Larva of *Hypena* (family Erebidae) on a leaf; **B**. Butterflies of the family Lycaenidae visiting flowers. Photos by K. Goula (**A**) and K. Touloumis (**B**).

The species grows in rock fissures, open stony places, or among small shrubs of *Juniperus oxycedrus* L. (Fig. 1), on basaltic and granitic substrates (Institute for Geology and Subsurface Research 1972; Tsikouras and Hatzipanagiotou 1995). Around the summit of Mt. Saos, it occurs together with other endemic or noteworthy taxa, such as *Drymocallis halacsyana* (Degen) Kurtto & Strid, *Galium samothracicum* Rech.f., *Herniaria degenii* (F. Herm.) Chaudhri, *Polygonum icaricum* Rech.f., *Stachys leucoglossa* subsp. samothracica (Degen) Biel & Kit Tan, and *Viola samothracica* (Degen) Raus. Although the flowers of *Vincetoxicum samothracicum* appear almost closed (Fig. 1B), they are frequently visited by butterflies of the family Lycaenidae (Fig. 7B), indicating that the distal upper part of the corolla is open. These butterflies may serve as pollinators. *Vincetoxicum samothracicum* is apparently a host to larvae of the genus *Hypena* Schrank (Erebidae; Fig. 7A), as also reported for the closely related species *V. canescens* and *V. tmoleum* (Wagner and Fritsch 2017).

##### Etymology.

The epithet “*samothracicum*” refers to the Greek name of the island, Samothraki, on which the new species occurs.

##### Conservation status.

*Vincetoxicum samothracicum* has a very limited range, with an extent of occurrence (EOO) of 1.991 km^2^ and an area of occupancy (AOO) of 16 km^2^, meeting the thresholds for Critically Endangered and Endangered, respectively, under IUCN criteria B1 and B2 (IUCN Standards and Petitions Committee 2024). However, the island population is estimated at about 2000 individuals, and the species does not appear to face any significant threats at present. Despite severe grazing pressure from numerous goats that ravage the flora of Mt. Saos, *V. samothracicum* remains unaffected, apparently due to toxic chemicals in the plant, as reported for other species of the genus (Prameela et al. 2024). The entire distribution range lies within the GR1110004 NATURA 2000 site and is also included in the national network of Roadless Areas (PAAs) as part of the Greek Ministry of Environment and Energy’s “Roadless Mountains” initiative, which aims at the strict protection of natural areas without road access (Ministry of Environment and Energy 2021). Considering all the above, the species is assessed as Least Concern (LC; IUCN Standards and Petitions Committee 2024). Monitoring of the species is highly recommended due to its very limited distribution. A potential future change in the mountain’s protection status, such as permitting the construction of wind farms, may result in the destruction of the species’ restricted distribution area.

### Key to the species of *Vincetoxicum* in Greece

**Table:** 

1	Corona lobes free almost to their base; corolla adaxially hirsute or lanate	**2**
–	Corona lobes at least partially fused; corolla adaxially glabrous to pubescent	**4**
2	Corolla and follicles reddish-purple; upper leaves lanceolate; corolla adaxially lanate	***V. samothracicum***
–	Corolla dull yellow, follicles greenish-yellow to green; upper leaves mostly ovate to ovate-lanceolate; corolla adaxially hirsute	**3**
3	Plant grayish tomentose, follicles gray puberulent (East Aegean Islands)	***V. canescens***
–	Plant subglabrous, follicles minutely puberulent to glabrescent (Kriti)	***V. creticum***
4	Corolla yellow or whitish	**5**
–	Corolla brown to brownish-purple, or dark purple	**6**
5	Leaves broadly ovate to ovate-lanceolate, acute, pubescent; calyx lobes linear; corolla glabrous adaxially	***V. hirundinaria***
–	Leaves ovate-lanceolate, long-acuminate, veins and margins ciliate; calyx lobes oblong; corolla with straight hairs adaxially	***V. huteri***
6	Corolla pubescent adaxially; plants velutinous	***V. speciosum***
–	Corolla glabrous; plants subglabrous or pubescent	**7**
7	Cymes glomerate or capitate, distinctly pedunculate, with numerous flowers; pedicels 1–3 mm long; corolla dark purple	***V. funebre***
–	Cymes 3–6 flowered, sessile; pedicels 5–10 mm long; corolla brown to brownish-purple	***V. fuscatum***

## Supplementary Material

XML Treatment for Vincetoxicum samothracicum

## Additional information

### Use of AI

No use of AI was reported.

### Author ORCIDs

Katerina Goula https://orcid.org/0000-0001-9207-3570

Theophanis Constantinidis https://orcid.org/0000-0001-9704-3864

### Data availability

All of the data that support the findings of this study are available in the main text or Supplementary Information.

## Supplementary materials

Supplementary material 1 Accession numbers Data type docx Explanation note List of taxa and their corresponding GenBank accession numbers used in the phylogenetic analyses. https://binary.pensoft.net/file/1522291This dataset is made available under the Open Database License (http://opendatacommons.org/licenses/odbl/1.0/). The Open Database License (ODbL) is a license agreement intended to allow users to freely share, modify, and use this Dataset while maintaining this same freedom for others, provided that the original source and author(s) are credited. Katerina Goula, Panagiotis Doumas, Theophanis Constantinidis

Supplementary material 2 Phylogenetic analyses Data type nex Explanation note The Nexus file used in the phylogenetic analyses. https://binary.pensoft.net/file/1522292This dataset is made available under the Open Database License (http://opendatacommons.org/licenses/odbl/1.0/). The Open Database License (ODbL) is a license agreement intended to allow users to freely share, modify, and use this Dataset while maintaining this same freedom for others, provided that the original source and author(s) are credited. Katerina Goula, Panagiotis Doumas, Theophanis Constantinidis
