# Supplementary material for: Vincetoxicum samothracicum (Apocynaceae), a new species from Mt. Saos, Samothraki Island, Greece
Source: PhytoKeys. 2026 Jan 28;270:41–56. doi: 10.3897/phytokeys.270.174922 (PMC12873569; doi:10.3897/phytokeys.270.174922)
Supplement: Supplementary material 1 — Accession numbers [file phytokeys-270-041_article-174922__-s001.docx]

**List of taxa and their corresponding GenBank accession numbers used in the phylogenetic analyses.**

| **Taxon** | **GenBank accession number** |
| --- | --- |
| *Cynanchum acutum* L. | MN106222.1 |
| *Gomphocarpos fruticosus* (L.) W.T.Aiton | AM396906.1 |
| *Vincetoxicum arnottianum* (Wight) Wight | LN880574.1 |
| *Vincetoxicum assadii* Zaeifi | LN880575.1 |
| *Vincetoxicum atratum* (Bunge) C.Morren & Decne. | HE793927.1 |
| *Vincetoxicum canescens* (Willd.) Decne. | LN880577.1 |
| *Vincetoxicum canescens* (Willd.) Decne. subsp. *canescens* (2) | MN106189.1 |
| *Vincetoxicum canescens* (Willd.) Decne. subsp. *canescens* (3) | MN106188.1 |
| *Vincetoxicum canescens* (Willd.) Decne. subsp. *canescens* (4) | MN106190.1 |
| *Vincetoxicum canescens* subsp. *pedunculata* Browicz (1) | MN106191.1 |
| *Vincetoxicum canescens* subsp. *pedunculata* Browicz (2) | MN106192.1 |
| *Vincetoxicum canescens* subsp. *pedunculata* Browicz (3) | MN106193.1 |
| *Vincetoxicum creticum* Browicz | HE793928.1 |
| *Vincetoxicum funebre* Boiss. & Kotschy (1) | LN880579.1 |
| *Vincetoxicum funebre* Boiss. & Kotschy (2) | MN106194.1 |
| *Vincetoxicum fuscatum* subsp. *boissieri* (Kusn.) Browicz (1) | MN106195.1 |
| *Vincetoxicum fuscatum* subsp. *boissieri* (Kusn.) Browicz (2) | MN106196.1 |
| *Vincetoxicum fuscatum* subsp. *boissieri* (Kusn.) Browicz (3) | MN106197.1 |
| *Vincetoxicum fuscatum* (Hornem.) Rchb. subsp. *fuscatum* (1) | MN106198.1 |
| *Vincetoxicum fuscatum* (Hornem.) Rchb. subsp. *fuscatum* (2) | MN106201.1 |
| *Vincetoxicum fuscatum* (Hornem.) Rchb. subsp. *fuscatum* (3) | MN106199.1 |
| *Vincetoxicum fuscatum* (Hornem.) Rchb. subsp. *fuscatum* (4) | MN106202.1 |
| *Vincetoxicum fuscatum* (Hornem.) Rchb. subsp. *fuscatum* (5) | MN106200.1 |
| *Vincetoxicum glaucum* (Wall. ex Wight) Rech.f. | LN880581.1 |
| *Vincetoxicum hirundinaria* Medik. (1) | MN106204.1 |
| *Vincetoxicum hirundinaria* Medik. (2) | MN106205.1 |
| *Vincetoxicum hirundinaria* Medik. (3) | MN106203.1 |
| *Vincetoxicum intermedium* Taliev | LN880583.1 |
| *Vincetoxicum jailicola* Juz. | LN880584.1 |
| *Vincetoxicum maeoticum* (Kleopow) Barbar. | LN880586.1 |
| *Vincetoxicum mozaffarianii* Zaeifi | LN880587.1 |
| *Vincetoxicum nigrum* (L.) Moench | FJ362532.1 |
| *Vincetoxicum parviflorum* Decne. (1) | MN106207.1 |
| *Vincetoxicum parviflorum* Decne. (2) | MN106206.1 |
| *Vincetoxicum pumilum* Decne. | LN880589.1 |
| *Vincetoxicum rehmannii* Boiss. | LN880590.1 |
| *Vincetoxicum rossicum* (Kleopow) Barbar. | FJ517165.1 |
| *Vincetoxicum sakesarense* Ali & Khatoon | LN880592.1 |
| *Vincetoxicum scandens* Sommier & Levier (1) | LN880594.1 |
| *Vincetoxicum scandens* Sommier & Levier (2) | MN106209.1 |
| *Vincetoxicum scandens* Sommier & Levier (3) | MN106208.1 |
| *Vincetoxicum scandens* Sommier & Levier (4) | MN106210.1 |
| *Vincetoxicum schmalhausenii* (Kusn.) Litv. | LN880595.1 |
| *Vincetoxicum speciosum* Boiss. & Spruner (1) | MN106214.1 |
| *Vincetoxicum speciosum* Boiss. & Spruner (2) | MN106211.1 |
| *Vincetoxicum speciosum* Boiss. & Spruner (3) | MN106212.1 |
| *Vincetoxicum speciosum* Boiss. & Spruner (4) | MN106213.1 |
| *Vincetoxicum stocksii* Ali & Khatoon | AJ320475.1 |
| *Vincetoxicum tauricum* Pobed. | LN880596.1 |
| *Vincetoxicum tmoleum* Boiss. (1) | LN880597.1 |
| *Vincetoxicum tmoleum* Boiss. (2) | MN106216.1 |
| *Vincetoxicum tmoleum* Boiss. (3) | MN106218.1 |
| *Vincetoxicum tmoleum* Boiss. (4) | MN106215.1 |
| *Vincetoxicum tmoleum* Boiss. (5) | MN106217.1 |
